# Catalysts of Change: Using AI To Lower the Activation Energy for Developing Gamified Learning Experiences in Health Profession Education

**DOI:** 10.1007/s40670-025-02635-x

**Published:** 2026-01-14

**Authors:** Stephanie N. Moore-Lotridge, Brianne E. Lewis

**Affiliations:** 1 Department of Orthopaedics, Vanderbilt University Medical Center, Nashville, TN USA; 2 School of Medicine, Vanderbilt University, Nashville, TN USA; 3 Vanderbilt Center for Bone Biology, Vanderbilt University Medical Center, Nashville, TN USA; 4 Department of Foundational Sciences, Central Michigan University College of Medicine, Mount Pleasant, MI USA

**Keywords:** Gamified Learning, Gamification, Generative AI, Educational Game, Escape Games, Serious Games, Health Professions Education

## Abstract

This monograph provides a practical guide for using generative AI, such as ChatGPT, to support the design and implementation of gamified learning in health professions education. By lowering the “activation energy” required to create immersive educational experiences, generative AI can help educators overcome common barriers such as available time, resources, and expertise. Here, we will examine a range of gamified learning strategies and illustrate how generative AI and prompt engineering can be used to support their design and implementation. While generative AI enhances creativity and workflow efficiency, faculty oversight remains essential to ensure accuracy, relevance, and alignment with educational goals.

## Introduction

Gamified learning, which refers to incorporating games, game elements (e.g., points, badges), or other playful, game-adjacent components into an educational environment, has emerged as a powerful and increasingly adopted strategy to transform health science education [1–3]. By fostering an immersive, motivating, and interactive learning environment, studies have shown that gamified learning can enhance learner’s satisfaction, while also leading to improvement in knowledge acquisition, critical thinking, problem-solving, and teamwork skills [4–7]. Likewise, gamified learning aligns with adult and experiential learning theories [7], making it a worthwhile and effective tool for developing practical competencies and clinical reasoning essential for modern healthcare professionals.

More specifically, gamified learning is grounded in multiple complementary learning theories [7–10]. Self-determination theory provides a well-established framework for understanding how game mechanics such as choice, progressive challenge, feedback, and collaboration enhance intrinsic motivation by supporting learners’ autonomy, competence, and relatedness [11, 12]. Experiential learning theory, particularly Kolb’s learning cycle [13], explains how gamified activities promote learning through cycles of action, reflection, and iteration. In parallel, cognitive load theory offers important guidance for gamified design, highlighting the need to manage intrinsic and extraneous load so that adaptive hints, feedback, and content generation support rather than overwhelm learners. Finally, social constructivist frameworks underpin the collaborative and team-based capacity of gamification strategies, emphasizing knowledge construction through interaction, shared problem-solving, and peer feedback. Together, these theoretical perspectives provide a cohesive rationale for how gamified learning strategies move beyond technical implementation toward theory-informed educational design [7–9].

However, health science educators face several challenges that can deter them from adopting gamified learning. These include financial constraints, lack of infrastructure and technical support, limited training and professional development, as well as negative perceptions and attitudes towards gamified learning [7, 14–16]. Broadly, these constraints and the “activation energy” necessary for the development of gamified learning have limited application in health professions education. However, with emerging tools such as artificial intelligence (AI), the inclusion of gamified activities is now more accessible, supporting a broader adoption of gamified learning in health professions education [15].

Thus, the goal of this manuscript is to provide practical tips for employing AI tools to support the development and implementation of gamified learning in health professions education. Here, we will provide practical guidance on how: (1) AI can support the selection of game types and mechanics to support specific learning objectives, and (2) illustrate how AI can assist in the creative process of designing captivating gamified elements to align with course content and learning objectives. While many examples in this work are focused on undergraduate medical education, prompts can be modified to support graduate medical education or continuing medical education for faculty.

## Starting Off

Before selecting game mechanics or diving into generative AI-supported design strategies, educators must first clarify the learning objectives and identify any non-movable contextual barriers.

### What Are Your Objectives?

As you begin to develop a gamified learning approach, it is important to first identify the learning objectives and content you wish to reinforce. Not all topics or learning objectives covered as part of health science education will lend themselves naturally to gamified learning. For example, prior literature has demonstrated the success of gamified learning within foundational sciences, such as biochemistry, pharmacology, and toxicology, physiology, radiology, and system-based practices (i.e. work-flow) [1, 2, 17–30]. While results evaluating learning outcomes from gamification and game-based learning approaches are heterogenous [10, 31], it has been demonstrated across studies that these approaches support or improve other measurements such as teamwork, collaboration, motivation, and immersion [32–37].

### What Are the Non-movable Barriers?

Furthermore, it is also critical to consider any “non-movable” barriers that may exist in your learning environment, which can impact the use of gamified learning broadly and the selection of gamified strategies. For example, considerations should include:

1. Student Time: How long do you have within your curriculum to implement the gamified intervention? Do you have hours available to implement pre-work?
2. Faculty Time: How much time/effort do you or your team have available to develop a gamified intervention? How much time/effort do you have available to moderate the gamified intervention?
3. Class Size: How many learners will be participating in the gamified intervention? Will this be done at the same time or asynchronously?
4. Curricular constraints: Does this learning activity need curricular approval? How does this activity align with the institution’s educational goals? Will you need to provide rational/a pitch to leadership?
5. Space: Will students be in-person or participating virtually? If in person, how is the physical space arranged where you will be conducting the gamified intervention?
6. Resources: What tools/resources do you have available to support your gamified intervention? What technology do you have available for both development and implementation within the learning environment?

## Up Next: What Strategies for Gamified Learning Should I use?

**Fig. 1:**
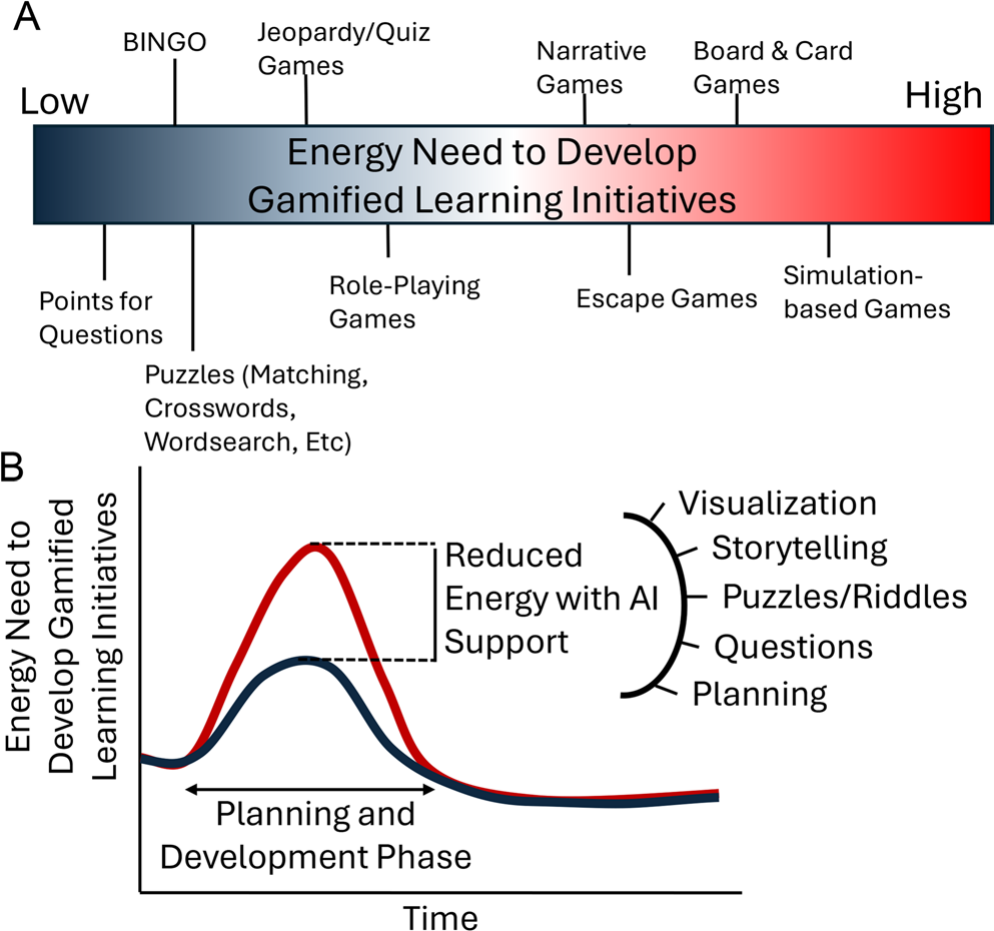
Generative AI can be used to support the lowering of the “Activation Energy” needed for the development and implementation of gamified learning initiatives. This monograph will explore how AI can support the planning and creation of a variety of gamified strategies. (**A**) Examples of gamified interventions ranging in activation energy needed for development. (**B**) Red line indicates the elevated energy needed during the development and implementation phase of a gamified learning strategy. Black line indicates the same process with the support of generative AI, resulting in a reduced energy expenditure. X axis indicates time to develop a gamified learning initiative, with the peak energy needed during development and implementation

Gamified learning in health professions education encompasses a range of strategies that markedly vary in “activation energy” needed to develop and implement (Fig. 1A). Independent of the strategy employed, the use of AI tools has the potential to lower the needed “activation energy” to make the development and implementation of gamified learning accessible to more faculty in health professions education (Fig. 1B).

To jumpstart the creative process, AI tools such as ChatGPT, can be useful to brainstorm the type of gamified elements that can be applied for a particular use case. For example:

### Example Prompt

I am a professor teaching biochemistry to 1 st year medical students in the United States. Please give me some ideas for how I could implement gamified learning into my lectures about GPCR signaling. I would like 3 ideas ranging in the amount of time and resources needed to implement them in a classroom (Low, Moderate, and High Energy Investiture).

### Example Response (ChatGPT-5)

Absolutely! Here are three tiered gamification strategies you could use for teaching GPCR signaling to first-year medical students, ranging from quick, low-prep add-ons to high-investment, reusable modules:

1. ***Low Energy Investiture: “GPCR Signaling BINGO”***
  *Time & Setup: 10–15 minutes; needs printable BINGO cards (you can reuse them yearly)*
  *How it Works: Create BINGO cards with terms related to GPCR signaling (e.g., Gαs, Gαi, adenylyl cyclase, β-arrestin, desensitization, phospholipase C, cAMP, DAG, etc.). During your lecture, as you teach or review the material, students mark off terms as they’re mentioned. Call out a few definitions or scenarios instead of just saying the terms (e.g., “This molecule activates PKA”→ they mark cAMP). Offer small prizes or recognition for first BINGO or “blackout” if you have time.*
  *Why It Works: Encourages active listening, reinforces vocabulary and concepts, and adds a fun, competitive element without disrupting the flow of lecture.*
2. ***Moderate Energy Investiture: “GPCR Role-Play Relay”***
  *Time & Setup: 30–45 minutes; requires printed character role cards or simple props.*
  *How it Works: Assign students roles (e.g., ligand, GPCR, G-protein subunits, adenylyl cyclase, cAMP, PKA). Students act out the signaling cascade, passing “messages” (like foam balls or index cards) that represent molecules. Include variations: inhibitory GPCRs, desensitization by arrestins, or different tissue-specific outcomes. Debrief with a diagram on the board and have students map their “performance” onto the pathway.*
  *Why It Works: Kinesthetic learning and storytelling reinforce complex sequences and molecular interactions.*
3. ***High Energy Investiture: “GPCR Escape Room: Cellular Distress!”***
  *Time & Setup: 60–90 minutes; needs puzzle design, printed materials, small props/locks or digital tools.*
  *How it Works: Students enter a narrative: “A cell is under attack, and only by restoring proper GPCR signaling can it survive.” Teams solve a sequence of puzzles that simulate GPCR-related challenges (e.g., determine the outcome of a signaling error, decode a message using cAMP concentration, match ligands to receptors). Each puzzle unlocks the next phase of the signaling cascade. Include QR codes or videos for multimedia clues.*
  *Why It Works: High engagement, team-based problem-solving, and application of knowledge in context. This also encourages metacognitive reflection after the activity.*

From these recommendations, faculty experts must select the gamified strategies that best align with the cognitive demands of the learning objective (e.g., recall versus application), the desired level of social interaction (teamwork or solo), and the approach that best aligns with the previously identified “non-movable barriers”.

## Your AI Assistant: Bringing your Gamified Learning Strategies To Life

After selecting a form of gamified learning that aligns with your environment, course content, and learning objects, the next hurdle faculty face is the design and implementation. In this section, we will illustrate how AI can assist in the creative process of designing and implementing captivating, gamified elements. We will specifically highlight low, moderate, and high energy investiture strategies, with a focus on how AI can help lower the needed “activation energy” to design and implement, including prompt engineering.

### Low Energy Investiture Activities

**Fig. 2:**
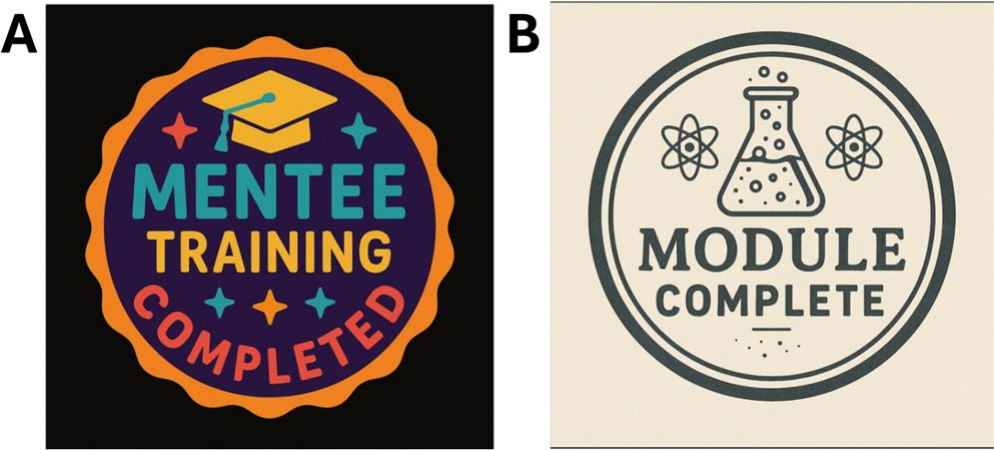
**A **& **B**) Illustrations of two “badges” generated with the use of generative AI (Copilot)

**Fig. 3:**
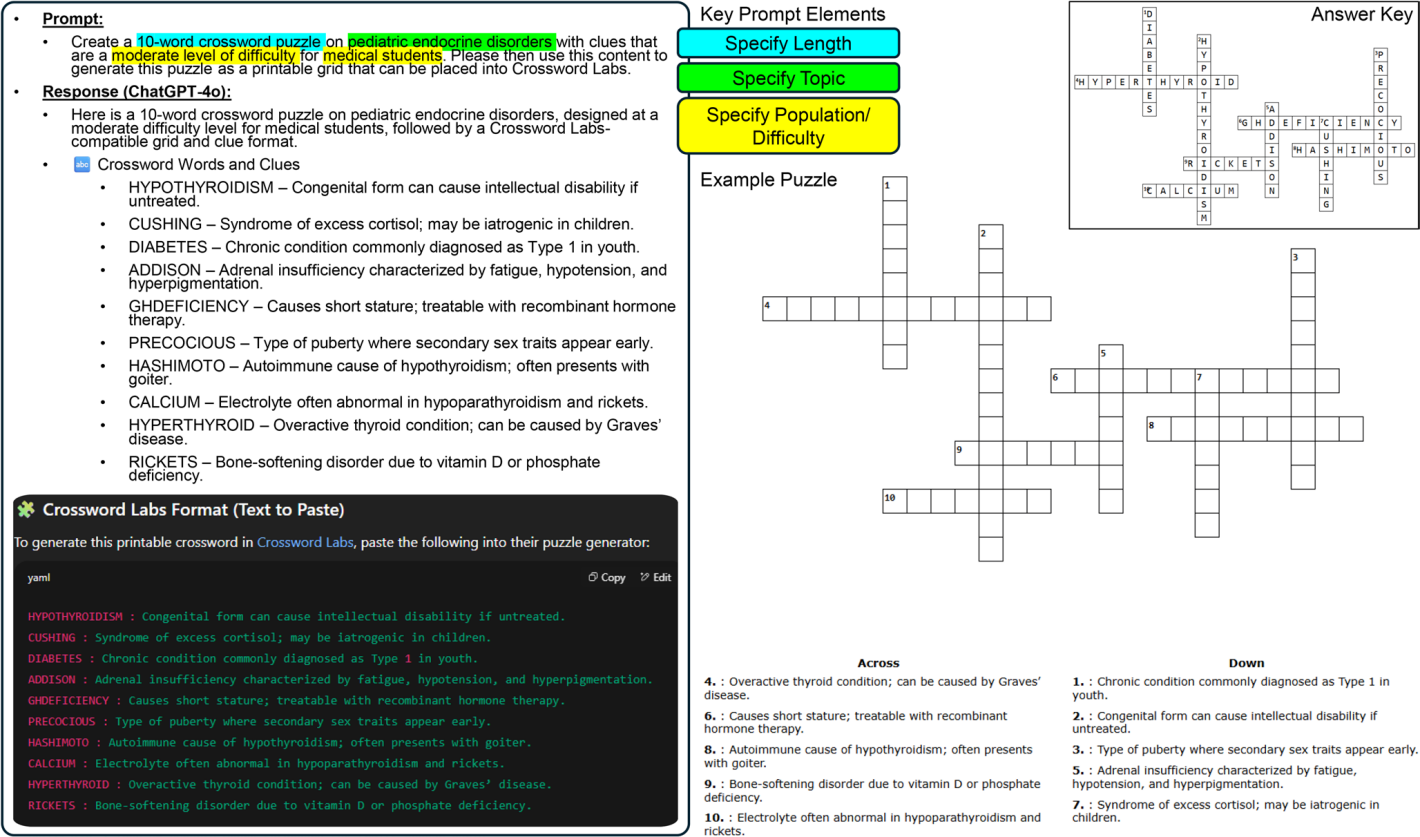
Representation of prompt, generative AI output, and crossword puzzle created. A prompt containing key design specifics, including puzzle length, topic of interest, and difficulty for a specific student population was entered into ChatGPT-4o. The response was then copied and entered into Crossword Labs to generate a puzzle as viewed, along with an answer key

**Fig. 4:**
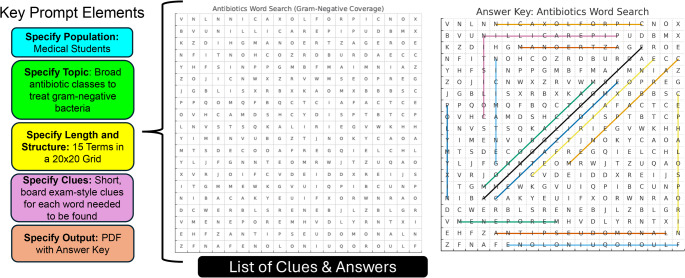
Representation of key prompt elements and wordsearch outputs, including a list of clues with answers (not shown) and answer key for wordsearch. Output shown here is generated with ChatGPT-4o

Gamified learning strategies that require a relatively low number of resources (energy and materials) to design and implement include digital badge that reward progress and mastery of competencies, crossword puzzles, wordsearches, bingo, or matching games to reinforce content. Generative AI can support these gamified learning initiatives by supporting development and implementation in the following ways:

1. **Development of Visuals for Badges**: Introducing badges into your teaching adds an element of gamification. Faculty may not have time or graphic design skills to rapidly generate customized badges to use in their teaching. In these cases, generative AI tools such as Copilot and ChatGPT can be used to generate and customize images to reward student progress and mastery of content. Badges can then be presented to students either in a physical form or through a digital platform such as a google sheets or your institutions learning management system to add interesting visuals and reward systems. An example of badges made with Copilot is shown in Fig. 2.

- *Example prompt: Design me an image of an achievement badge to be used for a mentee who has completed their mentee training. Make it with a black background with playful colors. (Fig.* 2 *A)*
- *Example prompt: Design me an image of an achievement badge to be used for students who have completed their biochemistry module. Make it black and white with scientific elements. (Fig.* 2 *B)*

2) **Development of Crossword Puzzles**: Generative AI tools can be used to generate themed crossword clues and puzzles for use in health science education. Prompt engineering can be used to direct the content, the relative difficulty, and the size of the puzzle.
  - *Example prompt: “Create a 10-word crossword puzzle on pediatric endocrine disorders with clues that are a moderate level of difficulty for medical students. Please then use this content to generate this puzzle as a printable grid that can be placed into Crossword Labs.”*
  - Online tools such as crosswordlabs.com can be used in tandem with content generated by AI to produce a functional product that can be printed or shared digitally with a link or embedded within your learning management system (Fig. 3).

3) **Development of Wordsearch with clues**: Generative AI is likewise an excellent tool for creating word searches with clues—a more educational and engaging twist on the classic word search format. For example, this form of gamified learning can be useful in situations where learners must link terminology with definitions or clinical features. Generative AI can be used to generate themed word lists based on a specific topic and accompanying clues that can be exported as a PDF to print or digitally share with students. Alternatively, this information can be formatted to use in online tools such as WordSearchLabs or PuzzleMaker to generate the puzzle (Fig. 4).

- *Example prompt: Create a word search puzzle for medical students focused on broad antibiotic classes and specific antibiotics that treat gram-negative bacteria. Include a list of 15 terms: a mix of broad antibiotic classes and specific antibiotics that treat gram-negative bacteria (e.g.*,* ciprofloxacin*,* gentamicin*,* meropenem). Instead of showing the word list*,* provide short*,* board-exam-style clues for each word*,* as students must solve the clues to find the words in the grid. Present the full word search grid (20 × 20) with all 15 terms hidden in various directions (forward*,* backward*,* vertical*,* diagonal). Then*,* generate an answer key graphic showing the location of each word within the grid. Finally*,* compile the puzzle and clues into a print-ready PDF with two pages: (1) the word search grid and (2) the clue list. Ensure the difficulty is appropriate for medical students studying pharmacology or infectious disease.*

4) **Development of Terminology Bingo**: Generative AI tools can be used to generate ready-made bingo cards for use in conjunction with a lecture, review session, or stand-alone activity. As highlighted above, bingo cards can be created with terms related to the content of interest. Students can then mark squares off as they are discussed or as 2nd order clues are provided (e.g., “This molecule activates PKA” → they mark cAMP).
  - *Example Prompt: I’m a professor teaching first-year medical students*,* and I’d like to create a classroom BINGO game to reinforce learning on the topic of nutrition. Please do the following: Generate a 5 × 5 BINGO card using 24 unique key terms*,* phrases*,* or concepts relevant to nutrition (e.g.*,* micronutrients*,* metabolism*,* dietary deficiencies*,* clinical syndromes*,* regulatory hormones*,* etc.). The center square should be labeled “FREE.” Provide a numbered list of corresponding clues or short definitions that match each of the 24 terms. These will be read aloud during the game. After generating the initial card*,* create 20 additional BINGO cards using the same 24 terms (plus the FREE space)*,* but with the terms randomly shuffled in each version to ensure that all cards are unique. All cards should be made in landscape format. Export each BINGO card as an individual print-ready PDF file*,* labeled “Bingo_Card_1.pdf*,*” “Bingo_Card_2.pdf*,*” etc. Please format each card clearly for classroom use.*
5) **Development of Matching Games**: Generative AIcan be used to help develop matching games for health professional students. Specifically, this modality can be useful for reinforcing terminology, disease presentations, drug mechanisms, and anatomy. Additionally, the difficulty of this activity can be tailored with the help of generative AI by including clue-based definitions instead of direct names, including distractors (i.e. extra terms that don’t match), or requiring two step logic (Match the hormone Disorder Symptom). This gamified learning tool can then be exported directly as a printable worksheet or imported into an online tool such as Flippity Matching Game, Educandy, or Wordwall.
  - *Example Prompt: Create a printable matching game for medical students focused on renal physiology. The difficulty should be moderate*,* suitable for learners who have basic knowledge of nephron function*,* transporters*,* and fluid/electrolyte regulation. Requirements: Create two columns: Column A (Terms/Scenarios) and Column B (Definitions/Explanations). Include 12 items in Column A*,* with 15 items in Column B (i.e.*,* 3 distractors that don’t match anything). Include a mix of single-step matches (e.g.*,* “Loop of Henle” → “Site of sodium reabsorption”) and two-step reasoning (e.g.*,* “Aldosterone effect” → “Increases sodium reabsorption and potassium secretion in the distal nephron”). Ensure terminology is specific to renal physiology (e.g.*,* GFR*,* ADH*,* aquaporins*,* RAAS*,* transporters). Provide a clear layout suitable for printing*,* ideally in a table format or labeled list (e.g.*,* A–L and 1–15). Please make this a visual layout for printing that can support matching with lines between correct answers as a PDF. In addition*,* provide an answer sheet as a PDF that can be provided to students after the activity.*

### Moderate Energy Investiture Activities

Gamified learning strategies that require a moderate amount of energy (time and resources) to design and implement include competitive quiz games, point-based learning modules, and serious games developed from existing popular game mechanics. Generative AI can support these gamified learning initiatives in the following ways:

1. **Competitive quiz games**: Competitive quiz games are some of the most common gamified learning strategies employed in health science education to promote active recall and friendly competition. Generative AI tools have been demonstrated in multiple studies to support the design of multiple-choice questions (MCQs) [38–40], though it is important to note that faculty review is necessary to ensure content accuracy [41]. As highlighted by Stadler et al. and others, best practices include rigorous review of all MCQs against authoritative sources, given the possibility of generative AI to “hallucinate” responses [41–43].
  - *Example Prompt for Question Generation: I’m designing a quiz game for first-year medical students. Please generate 10 multiple choice questions (MCQs) and 10 true/false questions on the topic of cardiovascular physiology. The questions should be: (1) At an appropriate difficulty level for first-year medical students. (2) A mix of conceptual understanding and clinical relevance. (3) Each question should have one correct answer and four plausible distractors. (4) Provide the correct answer and a brief explanation for why it’s correct. (5) Avoid overly obscure trivia or memorization-based questions. (6) Provide difficulty tags for the questions to support a point-based system.*
  - *Example Prompt for NBME-Style Question Generation: Generate a USMLE-style multiple-choice question that reflects the format and cognitive rigor of NBME Step 1 or Step 2 exams. The question should include a clinical vignette (3–5 sentences) with a clear stem and lead in question (e.g.*,* “Which of the following is the most likely diagnosis?“). It must include one correct answer and 4 plausible distractors. Questions should emphasize application of knowledge*,* not simple recall. The question should be appropriate for a 2nd year medical student*,* focused on fracture healing. Please include a brief explanation of the correct answer and why the distractors are incorrect.*

In addition to helping generate content for competitive quiz games, generative AI tools such as ChatGPT can be used to support the brainstorming of game formats, scoring rules, progression systems, and thematic branding to promote student engagement.

iii. *Example Prompt for Game Design: Create a competitive team-based quiz game for 1 st year medical students about cardiovascular physiology. Include rules*,* a point system*,* a game board*,* and a theme that will promote student engagement. Give me suggestions for how this activity can be run in class. Question format will include multiple choice questions*,* true and false*,* image identification*,* and a strategic risk round.*

2) **Development of Point-Based Learning Modules**: Generative AI toolscan be used to help design and implement point-based learning modules including their design and assessment components. For example, generative AI can be used to help map core concepts with learning objectives, can help suggest point allocation schemes based on complexity and mastery level, and can provide suggestions on gamification elements such as the use of badges (see above), leaderboards, streak bonuses, or challenges with bonus points. Additionally, generative AI can be used to generate content such as MCQs, and multimodal formats (crosswords, flashcards, matching games, ect), which can be combined to form a learning module.
  - *Example prompt: I am a medical educator designing a point-based*,* gamified learning module on acid-base disturbances for 1 st year medical students. The goal is to create an engaging*,* structured learning experience that supports both self-directed learning and formative assessment. This will be done asynchronously outside of classroom time. Please help me by doing the following: Map key acid-base concepts (e.g.*,* normal values*,* metabolic vs. respiratory disturbances*,* compensation mechanisms*,* clinical relevance) to activities that I could use as part of this module such as interactive challenges*,* quizzes*,* case-based unlocks*,* badges*,* or timed tasks. Suggest a point-based structure for the module*,* with recommended tiers (e.g.*,* foundational*,* intermediate*,* advanced) and point values. Recommend a progression structure for learners that builds from basic to clinical application*,* and ideas for how to “level up” or earn bonus points. Include sample questions or activities (MCQs*,* clinical vignettes*,* matching tasks*,* etc.) at different difficulty levels to help me populate the module.*
3) **Serious games based on popular game mechanics**: Many educational board and card games have been developed by using mechanics and rules from popular commercial games as foundational elements. By adapting familiar formats and gameplay, educators can create meaningful learning experiences that mirror the excitement of traditional gameplay. Examples include: (1) Dr. Jargon which uses game mechanics similar to taboo where players must get their team mate to guess a specific work of phrase without using certain related “Forbidden” words listed on the card, (2) Med Pursuit which uses game mechanics similar to trivial pursuit where students move around a board while answering category-based questions, and (3) “Medical Monopoly” which uses monopoly style game mechanics to teach about the healthcare system, hospital management, and ethical decision making. AI can be useful in the development of these serious games based on popular game mechanics in a variety of ways including:

- *Development of questions and content*: As highlighted above, generative AI can be useful in generating a variety of question types including MCQ, true/false, riddles, and fill in the blank questions that can be used as part of game play. Through prompt engineering, results can be focused on specific content, difficulty, and style of question desired.
- *Brainstorming popular game mechanics & rules*: As highlighted when brainstorming broad examples of gamified learning strategies, generative AI can likewise be used to brainstorm popular game mechanics and rules that could be applied to a specific content area.
  1. *Example prompt: I am a medical educator developing a serious educational game for students. I would like to incorporate popular board or card game mechanics to make the game both engaging and educational. Please help me by doing the following: (1) List 5–7 mainstream board or card games that have well-known and engaging mechanics that could be applied to educational content. (2) Briefly explain the core mechanic(s) of each game listed. (3) For each game*,* suggest how that core mechanic could be adapted to teach or assess student knowledge in the following content area: clinical decision-making in infectious disease management (e.g.*,* choosing the right antibiotic for a patient scenario). (4) Include ideas for win conditions*,* point systems*,* or player progression that could align with the learning objectives*,* but do not stray from the original game. The goal is to generate brainstorming ideas for a serious game that incorporates meaningful learning with recognizable gameplay.*
- *Development of visualization*: Once you have selected a popular game mechanic on which you would like to model a serious game, generative AI tools such as Copilot, ChatGPT, or Midjourney can assist with the development of the visualizations necessary for gameplay. This can include design of game boards, character profiles, game cards, and accessory materials (money, player tokens, score cards, ect).

### High Energy Investiture Activities

Gamified learning strategies that require a high amount of energy (time and resources) to design and implement include escape rooms, simulation-based games, immersive narrative and role-playing games, and de-novo generation of serious games that move beyond established popular game mechanics. As seen above, generative AI can support these gamified learning initiatives by lowering the “activation energy” for development and implementation in the following ways:

1. **Escape Games**: Generative AI tools can be used in a variety of ways to support the design and implementation of escape games for use in health professions education. This can include brainstorming puzzle structures and game design as well as creating an immersive storyline in which puzzles can be framed. Example prompts for how elements of an escape game can be created with the support of generative AI are highlighted below:
  - *Example Prompt (Story Line & Puzzle Types): “I’m designing an educational escape room activity for 1 st year medical students to reinforce key concepts in biochemistry- including cell signaling and lipid membranes. I want the game to have a compelling story that draws learners in while aligning with our learning objectives. Please help me by brainstorming a few creative storylines or scenarios that meet the following criteria: (1) Educationally Relevant: The story should naturally incorporate core medical concepts*,* (2) High Stakes or Urgency: Escape games work best when the story involves time pressure or a goal. (3) Immersive & Engaging: Include setting details*,* characters*,* and the inciting incident that drives the puzzle-solving. (4) Puzzle-Embedded: The story should make space for solving 4–6 interconnected puzzles that relate to the medical content and build toward solving a final challenge.”*
  - Example Prompt (Puzzle components): To support the development of puzzles used as part of an escape game, generative AI can be helpful in generating full puzzles, or components such as riddles and clues. For example:
    1. Prompt for matching puzzle: *“Help me create a matching activity for an escape room where students must pair related items (receptors and signaling pathway). Include 6–8 pairs and describe how the puzzle should be presented (e.g.*,* cards*,* images*,* physical objects).”*
    2. Prompt for Lock-based puzzle: *“I’m designing an escape room activity for 1 st year medical students. Can you generate a lock-based puzzle (e.g.*,* number code or combination lock) that teaches or tests knowledge of lipid metabolism? Please include the puzzle instructions*,* the solution*,* and a brief explanation of how the puzzle reinforces the topic.*”
    3. Prompt for Riddles: *“Create a clever riddle that leads students to identify a key term*,* “phospholipid”. The riddle should be challenging but solvable by 1 st year medical students with basic knowledge of the subject. Include the riddle*,* the answer*,* and a short explanation.”*
  - Visualization: Once a storyline and puzzles are established, generative AI can be used to generate visualizations to accompany the escape game to improve the immersive nature of the activity. This can be easily done by providing sections of the storyline to a generative AI tool and asking the system to produce a visualization in the style of interest (colorful, black and white, gothic, ect). This can then be repeated throughout the storyline to visualize the escape game.
  - An example of a biochemistry escape game created by the authors using the above strategies can be found at: https://view.genially.com/686fc8f03781c284602efd90. Importantly, this escape game was run in the first iteration by printing all materials and providing them to students as they worked in small groups. To increase usability and limit resources needed, it was transitioned to a digital format for the 2nd year which can be viewed above.
2. **Simulation-Based Games**: Simulation-based games are utilized in health science education to provide learners with immersive, experiential environments to practice clinical reasoning, teamwork, and decision-making without real-world consequences. These games replicate authentic patient care scenarios, enabling safe exploration of complex or rare cases. Generative AIcan be used to support the development of simulation-based games in a variety of ways, including generating patient presentations and helping to tailor the difficulty of the activity to the learner. Furthermore, AI powered “chat bots” have now been developed to serve as standardized patients, enabling real-time feedback through natural language processing and adaptive learning algorithms. As such, numerous reports for the use of AI in developing simulation-based games in health science education have been published [44].
3. **Immersive Narrative and Role-Playing Games**: In medical education, immersive narrative and role-playing games (RPGs) are interactive learning approaches that engage students through storytelling and character-driven scenarios. Much like the use in escape games, generative AI can support a multitude of steps during the design and implementation of these gamified learning strategies including brainstorming, storyline development relative to learning objectives, character development, and visualization.

Immersive narrative games place learners in a rich, story-based environment where educational content is embedded within a compelling plot. These narratives can be realistic (e.g. simulate real-life medical dilemmas) or imaginative (e.g. fantasy or science fiction-themed), often unfolding over time, and requiring learners to make decisions and reflect on outcomes. Generative AI can be utilized to help develop immersive storylines and mapping various routes learners may take in relation to their decisions. Furthermore, generative AI can be used to develop visualizations to accompany the storyline, as highlighted above for the development of escape games.

- *Example Prompt: I am a medical educator designing an interactive*,* narrative-based learning game for 1st-year medical students. Please generate a branching*,* decision-based storyline that allows learners to make choices that affect the outcome. The game should: (1) Focus on the topic of global health and infectious disease*,* specifically tuberculosis*,* and highlight challenges with resources and medication availability. (2) Be structured in 3–5 stages*,* each representing a step in clinical reasoning (e.g.*,* patient intake*,* history and physical*,* ordering labs*,* interpreting results*,* final diagnosis). (3) Include 3–4 decision points in each stage that branch into different pathways and outcomes (e.g.*,* correct vs. incorrect tests*,* missing clues*,* escalating symptoms). (4) Lead to multiple possible endings based on the learner’s choices—some optimal*,* some suboptimal*,* and some critical failures—with explanations for each outcome. (5) Integrate embedded teaching moments*,* such as short text blurbs or clues explaining why a decision was good or poor. Please generate a brief narrative game summary and title*,* a narrative outline of each stage with branching decision paths*,* examples of learner choices and consequences*,* and suggested feedback text or educational messages for each outcome.*

RPGs involve learners taking on specific characters in a simulated situation. This can likewise include realistic (healthcare) or imaginative settings depending on the content and learning objectives to be covered. These games emphasize communication, empathy, and ethical reasoning, and have been used to practice interprofessional collaboration or difficult conversations (e.g., breaking bad news, informed consent [45–49]. Generative AI can support the development of RPGs by supporting character development and associated images that can be included as part of larger narrative games.

b. *Example prompt: “I’m designing a narrative-based educational game for first-year medical students focused on community health outreach that includes role-playing elements. Please generate a diverse set of 6–8 realistic and engaging characters that students can act as (role-play) throughout the game. Each character should have: 1) A name*,* age*,* and brief background. 2) Their role within the healthcare or public health system (e.g.*,* patient*,* nurse*,* physician*,* community health worker*,* pharmacist*,* policy maker)*,* 3) Key personality traits and communication style*,* 4) A unique perspective or challenge related to the game’s topic*,* and 5) Potential ways they might influence the narrative outcome. Characters should reflect a range of cultural*,* socioeconomic*,* and professional backgrounds to enhance realism and empathy-building in the gameplay.”*

In summary, this manuscript provides a practical, theory-informed guide for health professions educators seeking to design and implement gamified learning with the support of generative AI. Through structured examples spanning low-, moderate-, and high-energy gamified strategies, the paper demonstrates how generative AI can support ideation, content generation, visualization, and implementation across diverse educational contexts. While examples focus primarily on undergraduate medical education, the strategies and prompts presented are adaptable to graduate medical education and continuing professional development, positioning gamification as an accessible and scalable approach to enhance engagement and learning in health professions education.

## Limitations

### Learner and Educator Perceptions and Adoption Barriers

A key limitation of gamified learning approaches in education relates to both learner and educator perceptions that influence adoption and sustainability [50–53]. Medical students are often highly examination-oriented, with a strong focus on standardized assessments such as STEP 1 and 2 in the United States. When gamified activities are not explicitly aligned with assessed content or learning objectives, learners may perceive them as inefficient, frivolous, or peripheral to exam preparation [54, 55]. Acceptance improves when the relevance to course objectives and examinations is made explicit, expectations are clearly communicated, and activities respect learners’ time constraints. From the educator’s perspective, gamification may be perceived as time-intensive, technically complex, or pedagogically risky, particularly in environments with limited protected time, minimal instructional design support, or high curricular density [50–53]. While generative AI can help alleviate some of these challenges, faculty may also express concern about maintaining academic rigor and aligning activities with assessment standards. Poorly scaffolded gamified interventions, excessive cognitive load, or unclear implementation strategies can reinforce skepticism among both learners and educators. Recognizing these parallel barriers is essential, as successful gamified learning requires intentional design that accounts for learner priorities and assessment pressures as well as educator workload, resources, and confidence in the instructional approach.

### Generative AI use in Development of Gamified Learning

Despite its many advantages, the use of generative AI in developing gamified learning comes with important limitations. Most notably, AI-generated content may contain factual inaccuracies, outdated information, or subtle misconceptions, underscoring the need for expert faculty review throughout the design and implementation process. Additionally, generative AI lacks contextual awareness of specific learner needs, curricular integration points, and institutional constraints, which can limit the relevance or appropriateness of its output. Knowing this, we recommend beginning with identifying your objectives and non-movable barriers that can influence the success of a gamified learning strategy. With this foundation, such considerations can be included in prompt engineering to better tailor responses for your specific environment and goals.

Ethical considerations surrounding the use of generative AI in gamified learning extend beyond concerns of over-reliance and include the potential perpetuation of societal biases related to race, gender, and socioeconomic status embedded within training data. In medical education, these biases may manifest through patient vignettes that reinforce stereotypes, unbalanced clinical narratives, or visual representations that lack demographic diversity. While we propose that generative AI can support faculty during the educational design and implementation process, it does not replace the pedagogical expertise and critical oversight required for thoughtful instructional design. Educators must therefore take intentional steps to identify and mitigate bias, including explicitly prompting AI tools to generate diverse and representative scenarios, critically reviewing AI-generated content for implicit bias, and engaging diverse review teams during the development and implementation phase of a new gamified learning initiative. These strategies are essential to ensure that AI-supported gamified learning promotes equity, inclusivity, and educational rigor rather than inadvertently reinforcing existing disparities.

It is important to note that not all generative AI tools have been designed to excel at the same tasks. For example, while ChatGPT (OpenAI) excels at natural language generation and brainstorming ideas, tools such as Copilot (Microsoft), DALL·E (OpenAI), and Midjourney are excellent at generating images that be used throughout gamified learning strategies. Finally, generative AI is rapidly evolving, with ongoing advancements improving its accuracy, contextual understanding, and ability to tailor outputs to specific educational needs. This will mean that as new tools are developed, faculty must consider their own best practices to stay up to date to support their future educational practices.

## Conclusions

Generative AI has emerged as a powerful tool in health professions education. Here, we have discussed in depth how generative AI can lower the “activation energy” required to design and implement gamified learning. This democratization of design and implementation of gamified learning will allow for a future where such activities can align with curricular goals, learner needs, and institutional constraints. While faculty oversight remains essential to ensure content accuracy and pedagogical value, the creative and logistical support offered by generative AI has the potential to transform how we engage learners in foundational and clinical sciences. Ultimately, this shift opens new possibilities for creating immersive learning experiences and, promoting teamwork in the learning environment for the next generation of learners.
